# Subversion of Host Innate Immunity by Uropathogenic *Escherichia coli*

**DOI:** 10.3390/pathogens5010002

**Published:** 2016-01-04

**Authors:** Patrick D. Olson, David A. Hunstad

**Affiliations:** 1 Medical Scientist Training Program, Washington University School of Medicine, 660 S. Euclid Ave., Campus Box 8208, St. Louis, MO 63110, USA; olsonp@wusm.wustl.edu; 2 Departments of Pediatrics and Molecular Microbiology, Washington University School of Medicine, 660 S. Euclid Ave., Campus Box 8208, St. Louis, MO 63110, USA

**Keywords:** urinary tract infection, immune evasion, effectors, neutrophils, intracellular bacterial communities, filamentation, cystitis

## Abstract

Uropathogenic *Escherichia coli* (UPEC) cause the majority of community-onset urinary tract infections (UTI) and represent a major etiologic agent of healthcare-associated UTI. Introduction of UPEC into the mammalian urinary tract evokes a well-described inflammatory response, comprising pro-inflammatory cytokines and chemokines as well as cellular elements (neutrophils and macrophages). In human UTI, this inflammatory response contributes to symptomatology and provides means for diagnosis by standard clinical testing. Early in acute cystitis, as demonstrated in murine models, UPEC gains access to an intracellular niche that protects a population of replicating bacteria from arriving phagocytes. To ensure the establishment of this protected niche, UPEC employ multiple strategies to attenuate and delay the initiation of host inflammatory components, including epithelial secretion of chemoattractants. Recent work has also revealed novel mechanisms by which UPEC blunts neutrophil migration across infected uroepithelium. Taken together, these attributes distinguish UPEC from commensal and nonpathogenic *E.* *coli* strains. This review highlights the unique immune evasion and suppression strategies of this bacterial pathogen and offers directions for further study; molecular understanding of these mechanisms will inform the development of adjunctive, anti-virulence therapeutics for UTI.

## 1. Introduction

The human immune system faces a constant obligation to provide surveillance at epithelial surfaces and to detect and eliminate pathogenic microbes. Innate responses are triggered by ligation of pathogen-recognition receptors on and within epithelial cells and resident immune cells. These responses activate and recruit phagocytes, particularly macrophages and neutrophils (polymorphonuclear leukocytes; PMNs), which act to engulf and kill bacterial pathogens at the point of their arrival. Conversely, in order to establish infection and thwart immune detection and clearance, microorganisms of all types have evolved a variety of molecular mechanisms to attenuate and evade mammalian innate immunity (reviewed in [1,2,3,4], among many others). Even among Gram-negative bacteria, the mechanisms of these effects vary widely. In many model pathogens (e.g., *Salmonella*, *Vibrio*, *Yersinia*), effector proteins interrupting host cell signaling, kinase activation, cytoskeletal rearrangement, post-translational modification, and other processes are delivered to the target cell via type III secretion. Here, we will outline the distinct immune modulation strategies of uropathogenic *Escherichia coli* (UPEC), the chief cause of urinary tract infections [5,6]. Unlike other pathotypes of *E. coli* that modulate host responses (e.g., enterohemorrhagic or enteropathogenic), UPEC do not encode type III secretion machinery [7].

The ascending introduction of UPEC or other pathogenic bacteria into the mammalian urinary tract induces an inflammatory response, initiated primarily by activation of Toll-like receptors (TLRs). Stimulation of TLR4 by bacterial lipopolysaccharides (LPS) activates the NF-κB pathway, inducing the expression of cytokines including IL-6 and neutrophil chemoattractants such as IL-8 (CXCL1), which are measurable in the urine of mice and humans with UTI [8,9]. Increases in uroepithelial cyclic AMP, activated by TLR4-induced increases in intracellular [Ca^2+^], also result in NF-κB-independent augmentation of IL-6 and IL-8 expression [10]. TLR5 stimulation, responding to bacterial flagellin, can additionally contribute to acute inflammation during UTI [11,12]. Perpetuation of the neutrophil response may be driven by cytokines such as IL-17, which has an emerging role in bridging innate to adaptive immunity [13] and is a mediator of the innate response during experimental UTI [14]. The human cathelicidin LL-37, a small, cationic antibacterial peptide, is detectable in the urine during human cystitis, and mice deficient in its ortholog (CRAMP) demonstrate increased susceptibility to pyelonephritis [15], although it may paradoxically enhance bladder infection [16]. Multiple members of the defensin class of antimicrobial peptides are also produced locally during UTI [17,18], and the participation of these molecules in the host-pathogen conversation remains a fertile area for study.

A core result of the soluble inflammatory response is the recruitment of neutrophils to the bladder. Indeed, the detection of neutrophils in the urine is a diagnostic hallmark of human UTI. The phagocytic capacity of neutrophils plays a critical role in controlling UPEC UTI, as demonstrated in multiple studies [19,20,21,22,23,24]. UPEC is able to effectively colonize the bladder in the face of this highly inflammatory soluble and cellular response, first attenuating phagocyte recruitment and subsequently evading the activity of neutrophils in the bladder. The molecular strategies underlying these UPEC phenotypes are the focus of this review.

## 2. Immune Evasion Strategies of UPEC

### 2.1. The UPEC Intracellular Bacterial Community

**Figure 1:**
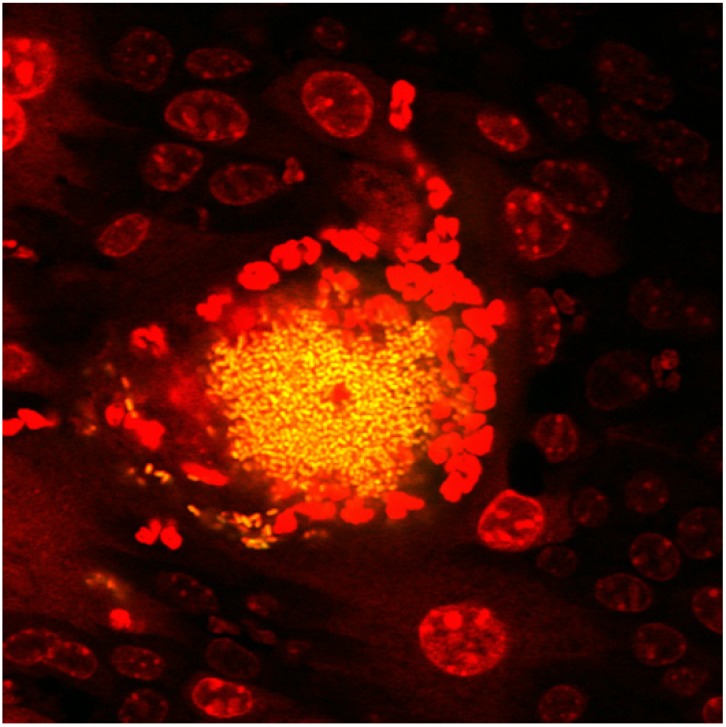
Intracellular UPEC communities are protected from neutrophil attack. Confocal microscopy of a C57BL/6J female mouse bladder harvested during acute cystitis (16 h post infection), viewed from a luminal perspective, shows an intracellular bacterial community of UPEC (yellow) within a binucleate superficial facet cell that is surrounded by recruited neutrophils (revealed by red nuclear staining; larger, round epithelial cell nuclei are also visible). UPEC strain UTI89 expressing green fluorescent protein (GFP) was used for infection, and bladder was fixed and stained with Syto 61 red nuclear stain [41].

As detailed elsewhere in this issue, acute UPEC cystitis relies on a well-documented intracellular pathogenesis cascade in which the pathogen is internalized into superficial bladder epithelial cells (also termed facet or umbrella cells) [25,26,27,28,29,30,31,32,33,34,35,36]. Despite comprising a minority of total bacteria within the infected bladder (most are luminal), intracellular UPEC coordinate a variety of essential functions to propagate infection. The facet cell cytoplasm provides a rich nutrient environment that supports the massive intracellular replication of UPEC in the acute phase of cystitis, forming the intracellular bacterial community (IBC) [30]. However, this locale also represents a vitally important, protected niche whereby UPEC can evade the phagocytic activity of arriving neutrophils. By video microscopy in the infected murine bladder, neutrophils can be observed engulfing luminal bacilli during the initial hours of infection [37]. However, bacteria that have successfully invaded uroepithelial cells are sheltered from this attack; neutrophils are clearly able to locate infected epithelial cells, but cannot access the expanding bacterial population comprising the IBC (Figure 1). As part of the host response to infection, many of the facet cells will ultimately be exfoliated into the urine—a means by which the host may rid itself of many thousands of bacteria. In order to escape this fate and perpetuate infection, a subset of UPEC must exit the IBC and initiate additional genetic programs that allow these bacteria to traverse the extracellular space in the face of a burgeoning luminal neutrophil population, thereby facilitating additional rounds of IBC formation in naïve epithelial cells. Importantly, the presence of both shed IBCs and filamentous *E. coli* has been demonstrated in the urines of women and children with acute cystitis [38,39,40].

### 2.2. Filamentation

**Figure 2:**
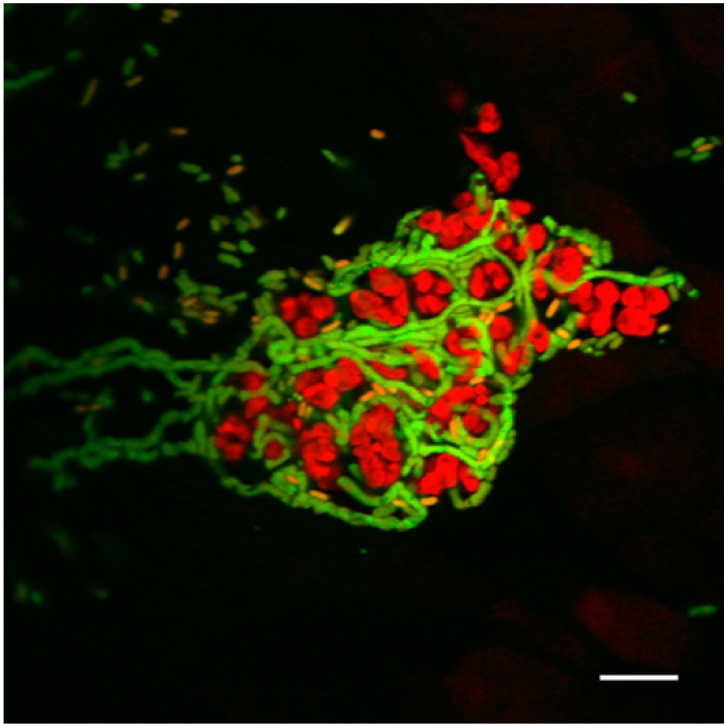
Filamentous UPEC resist phagocytosis in the bladder. Confocal microscopy of a C57BL/6J female mouse harvested 24 h post-infection shows a surface (luminal) collection of bacillary and filamentous GFP-expressing UPEC strain UTI89 (green) in which the bacterial filaments are heavily intermingled with neutrophils (red nuclei, as in Figure 1). Scale bar, 10 μm.

During maturation of the IBC, a minority of the intracellular population proceeds through a differentiation pathway that results in the formation of non-septated, filamentous bacteria up to 70 μm in length—a process that requires expression of the cell division inhibitor SulA [37,42]. Upon lysis of the IBC-harboring facet cell, UPEC flux out of the IBC proper to traverse the luminal surface. Video microscopy demonstrates that during this time of transition, bacillary forms of UPEC (1–2 μm in length) are readily engulfed, while filamentous UPEC resist phagocytosis, even when in direct contact with PMNs and macrophages [42,43] (Figure 2). At this juncture during murine cystitis, flow cytometric analysis of bladder luminal bacteria demonstrates enrichment of filamentous forms, consistent with a selection process in which bacillary forms are preferentially taken up by host phagocytes [37]. This preferential engulfment of standard bacilli is also supported by *in vitro* studies using cultured bladder epithelial cells and human macrophages [37,43]. As PMNs are in fact capable of engulfing particles (e.g., fungal hyphae) that are larger than themselves [44,45], the observed resistance of filamentous UPEC to PMN phagocytosis likely relies on attributes beyond the organism’s size [42]. Underscoring the pathogenic importance of filamentation, the UPEC *sulA* mutant forms a first round of IBCs without difficulty, but fails to sustain infection beyond this point, as it cannot filament in order to survive the luminal transition. Abrogation of the PMN response (e.g., in TLR4-deficient mice) rescues the *sulA* pathogenic defect [42].

The adoption of the filamentous phenotype by a subset of UPEC represents a response of the pathogen to stresses associated with the marshaling of host immune components. In support of this model, filamentation is not observed during UPEC UTI in TLR4-deficient hosts [42], which feature a sharply limited soluble and cellular inflammatory response to bacterial introduction. Meanwhile, filamentation can be induced *in vitro* by exposure of bacillary UPEC to LPS-activated macrophages [37,43]. Taken together, these observations demonstrate an elegant host-pathogen conversation taking place during this critical stage of UTI propagation.

### 2.3. UPEC Attenuation of Early Uroepithelial Cytokine Production

While the full extent of the neutrophilic response to bacterial cystitis is fully evident within hours, internalization of UPEC into bladder epithelial cells occurs initially in <1 h [30,31]. Substantial work by multiple groups has outlined a number of pathogenic strategies by which UPEC may dampen very early innate responses, effectively “holding off the cavalry” to protect luminal bacteria until the sheltered intracellular niche can be established.

Compared with commensal or laboratory strains of *E. coli*, UPEC display a marked ability to dominantly suppress epithelial cytokine production. UPEC stabilize IκB, thereby suppressing NF-κB activity and increasing bladder epithelial cell apoptosis [46]. Further, multiple UPEC isolates elicit lower levels of IL-6 and IL-8 from uroepithelial cells and are able to block secretion of these inflammatory cytokines upon co-inoculation with known NF-κB stimulators [47,48]. Genes involved in LPS biosynthesis (e.g., *rfa*, *rfb*, *waaL*) are also important for this phenotype, suggesting that UPEC LPS modifications may at least confer a less stimulatory LPS structure, or perhaps that a non-stimulatory LPS may exert a dominant effect [48,49]. In addition, lack of the OMP chaperone SurA abolishes the immunosuppressive phenotype [48]; reduced levels of the SurA-dependent LPS transporting protein LptD may alter the presentation of potentially non-stimulatory LPS [50].

However, LPS differences alone do not account for cytokine suppression [51], and it is likely that other mechanisms and UPEC effectors also contribute to suppressing cytokine secretion. For example, NF-κB signaling may be inhibited by sub-lytic concentrations of α-hemolysin (HlyA) [52,53], which is encoded on a UPEC pathogenicity island that also harbors *cnf1* (see Section 2.4). Exemplifying a distinct strategy, a substantial minority of UPEC isolates, including the pyelonephritis/urosepsis strain CFT073, encode a Toll/IL-1 receptor (TIR) domain-containing protein, termed TcpC, that interacts with the host adaptor MyD88 to inhibit TLR signaling [54]. TcpC disruption was associated with decreased bacterial burden in the kidneys and a reduction in histologically evident renal damage, indicating the importance of this protein during pyelonephritis. Further study of additional signaling pathways that rely on TIR domain-containing proteins (e.g., the TRIF and IL-6/IL-1 signaling cascades) revealed that TcpC also regulated these pathways independent of MyD88, suggesting that TcpC may impact pathogenesis in a broader way [55]. Finally, the SisA and SisB proteins, homologues of the immunomodulatory protein ShiA from *Shigella flexneri*, are found in CFT073 and have recently been implicated in immune modulation during UTI [56].

### 2.4. UPEC Inhibition of Neutrophil Recruitment and Function

Uropathogenic strains of *E. coli* feature additional virulence mechanisms targeting the recruitment and function of neutrophils. In contrast to commensal and laboratory *E. coli* isolates, UPEC are able to suppress neutrophil migration by down-regulating expression of many PMN genes involved in neutrophil chemotaxis, proinflammatory signaling, adhesion, and migration [57]. To study the molecular determinants of this phenomenon, we recently optimized an *in vitro* model of PMN migration across a cultured bladder epithelial cell monolayer [57,58,59]. UPEC isolates both attenuate transepithelial PMN migration *in vitro* and elicit reduced bladder PMN influx *in vivo*, in contrast to nonpathogenic strains [57,59].

UPEC have been shown to induce a number of anti-inflammatory molecules within PMNs and uroepithelial cells, including indoleamine 2,3-dioxygenase (IDO) [57]. IDO represents the first enzyme in the catabolism of tryptophan and is well known to modulate adaptive immunity, specifically T cell functions, by starving the local milieu of tryptophan [60]. Unlike nonpathogenic *E. coli*, UPEC specifically upregulate IDO locally within 1 h after introduction into the murine bladder, resulting in attenuation of innate cellular responses (*i.e.*, neutrophil recruitment). Compared with wild-type hosts, UTI in IDO-deficient mice features augmented PMN migration, corresponding with increased killing of extracellular (luminal) bacteria and attenuation of infection [61]. Though the key IDO-expressing cell types have not yet been specified, contributions from both the epithelial and hematopoietic compartments are being investigated. Independent of this question, the sequential influence of two canonical IDO stimulators, namely TNFα and interferons (both type I and IFNγ), appears to underlie UPEC induction of IDO in the host ([61] and unpublished data). Notably, IDO influence on PMN migration is not mediated via tryptophan starvation, as experimental addition of exogenous tryptophan does not rescue neutrophil migration. Instead, the products of tryptophan catabolism, known collectively as kynurenines, may exert a direct influence on PMN transuroepithelial migration.

UPEC also employ a variety of secreted toxins and effectors to neutralize the phagocytic activity of PMNs. As noted above, α-hemolysin (HlyA) is cytolytic to hematopoietic cells but also has more subtle immune-modulating effects at sub-lytic concentrations [62,63]. The genetically linked UPEC toxin termed cytotoxic necrotizing factor 1 (CNF1) suppresses PMN chemotaxis, phagocytic activity and release of reactive oxygen species [64,65]. Interestingly, CNF1 appears to be packaged into outer membrane vesicles, structures liberated by all Gram-negative bacteria, for delivery to target phagocytes [65]. A newly identified UPEC effector, termed YbcL, has been shown to inhibit transepithelial migration of human neutrophils *in vivo* and *in vitro* via a mechanism that is distinct but as yet incompletely defined. This effector localizes initially to the bacterial periplasm and is subsequently liberated via bacterial lysis, suppressing PMN transepithelial migration in both *in vivo* and *in vitro* model systems [59,66]. YbcL homologs are present in laboratory and commensal *E. coli* isolates but are unable to suppress PMN migration due to a single amino acid alteration, while most UPEC strains encode the suppressive YbcL variant [59]. Interestingly, YbcL suppression of PMN migration requires the presence of uroepithelial cells (*i.e.*, it does not act directly on PMN alone), and the bacterial lysis that liberates YbcL is augmented by bacterial exposure to bladder epithelia [66]. These observations highlight a form of bacterial altruism in which a subset of UPEC arriving in the mammalian bladder are sacrificed such that others in the nascent UPEC community may achieve success in colonizing the bladder epithelium.

## 3. Does Inflammation Ultimately *Promote* UPEC Infection?

A recent body of literature supports a model in which host inflammation during UTI may actually represent a double-edged sword. As has been discussed, the acute inflammatory response is essential for controlling UTI [20,67,68]; however, overly exuberant inflammatory responses during acute cystitis may be associated with increased tissue damage, predisposing the host to developing chronic forms of infection [69,70]. In susceptible murine hosts (e.g., C3H/HeN), a fraction of mice are unable to resolve acute UTI, developing long-term chronic bacterial cystitis with ongoing high tissue bacterial burden and inflammation. This appears to arise as the result of an early immune checkpoint, which can be altered experimentally by prior treatment with anti-inflammatory agents, subsequently preventing chronic infection [69,71]. Further, after chronic cystitis has become established, hosts are unable to resolve infection despite an intense, ongoing leukocyte response, and restoration of the exfoliated transitional epithelium is inhibited [69]. This inflamed state may thus allow UPEC access to more protected niches and deeper cell layers. Thus, hosts need to mount a finely tuned inflammatory response in order to eliminate uropathogens while avoiding the detrimental consequences of an exaggerated response that may augment disease severity or promote persistent infection.

## 4. Conclusions and Future Directions

Our knowledge of acute immunity during UTI and the mechanisms that UPEC exploit to evade it has increased dramatically over the past decade. Ongoing work aims to establish the molecular mechanisms of recently published immune evasion effectors and strategies. For example, previous studies suggest that YbcL has greatest structural homology to the mammalian Raf kinase inhibitory protein and may therefore interact with eukaryotic kinases [72]; specifying putative binding partners and targets within a host cell may elucidate the mechanism of this novel PMN trafficking inhibitor. In the same light, we are investigating how kynurenines generated by IDO-mediated tryptophan catabolism influence neutrophil migration. These studies will further illuminate the complex host-pathogen interactions occurring in the very early stages of UTI establishment.

Similarly, our understanding of protective immunity and the predictors of recurrent UTI is incomplete. Multiple groups are continuing work on the humoral immune response to cystitis and pyelonephritis and identification of immunodominant antigens. As well, studies of adaptive cellular responses with correlation to recurrence risk should be prioritized. In the decade to come, it is hoped that such investigations will enable novel and translatable approaches to limit the morbidity of these recalcitrant infections.
